# Whole-genome de novo sequencing reveals genomic variants associated with differences of sex development in SRY negative pigs

**DOI:** 10.1186/s13293-024-00644-w

**Published:** 2024-09-02

**Authors:** Jinhua Wu, Shuwen Tan, Zheng Feng, Haiquan Zhao, Congying Yu, Yin Yang, Bingzhou Zhong, Wenxiao Zheng, Hui Yu, Hua Li

**Affiliations:** Guangdong Provincial Key Laboratory of Animal Molecular Design and Precise Breeding, School of Life Science and Engineering, Foshan University, Foshan, 528255 P.R. China

**Keywords:** *SRY* negative pig, XX DSD, De novo sequencing, Single-nucleotide polymorphism, Structural variation

## Abstract

**Background:**

Differences of sex development (DSD) are congenital conditions in which chromosomal, gonadal, or phenotypic sex is atypical. In more than 50% of human DSD cases, a molecular diagnosis is not available. In intensively farmed pig populations, the incidence of XX DSD pigs is relatively high, leading to economic losses for pig breeders. Interestingly, in the majority of 38, XX DSD pigs, gonads still develop into testis-like structures or ovotestes despite the absence of the testis-determining gene (*SRY*). However, the current understanding of the molecular background of XX DSD pigs remains limited.

**Methods:**

Anatomical and histological characteristics of XX DSD pigs were analysed using necropsy and HE staining. We employed whole-genome sequencing (WGS) with 10× Genomics technology and used de novo assembly methodology to study normal female and XX DSD pigs. Finally, the identified variants were validated in 32 XX DSD pigs, and the expression levels of the candidate variants in the gonads of XX DSD pigs were further examined.

**Results:**

XX DSD pigs are characterised by the intersex reproductive organs and the absence of germ cells in the seminiferous tubules of the gonads. We identified 4,950 single-nucleotide polymorphisms (SNPs) from non-synonymous mutations in XX DSD pigs. Cohort validation results highlighted two specific SNPs, “c.218T > C” in the “Interferon-induced transmembrane protein 1 gene (*IFITM1*)” and “c.1043C > G” in the “Newborn ovary homeobox gene (*NOBOX*)”, which were found exclusively in XX DSD pigs. Moreover, we verified 14 candidate structural variants (SVs) from 1,474 SVs, identifying a 70 bp deletion fragment in intron 5 of the WW domain-containing oxidoreductase gene (*WWOX*) in 62.5% of XX DSD pigs. The expression levels of these three candidate genes in the gonads of XX DSD pigs were significantly different from those of normal female pigs.

**Conclusion:**

The nucleotide changes of *IFITM1* (c.218T > C), *NOBOX* (c.1043 C > G), and a 70 bp deletion fragment of the *WWOX* were the most dominant variants among XX DSD pigs. This study provides a theoretical basis for better understanding the molecular background of XX DSD pigs.

**Plain language summary:**

DSD are conditions affecting development of the gonads or genitalia. These disorders can happen in many different types of animals, including pigs, goats, dogs, and people. In people, DSD happens in about 0.02–0.13% of births, and in pigs, the rate is between 0.08% and 0.75%. Pigs have a common type of DSD where the animal has female chromosomes (38, XX) but no *SRY* gene, which is usually found on the Y chromosome in males. XX DSD pigs may look like both males and females on the outside and have testis-like or ovotestis (a mix of ovary and testis) gonads inside. XX DSD pigs often lead to not being able to have piglets, slower growth, lower chance of survival, and poorer meat quality. Here, we used a method called whole-genome de novo sequencing to look for variants in the DNA of XX DSD pigs. We then checked these differences in a larger group of pigs. Our results reveal the nucleotide changes in *IFITM1* (c.218T > C), *NOBOX* (c.1043 C > G), and a 70 bp deletion fragment in intron 5 of the *WWOX*, all linked to XX DSD pigs. The expression levels of these three genes were also different in the gonads of XX DSD pigs compared to normal female pigs. These variants are expected to serve as valuable molecular markers for XX DSD pigs. Because pigs are a lot like humans in their genes, physiology, and body structure, this research could help us learn more about what causes DSD in people.

**Supplementary Information:**

The online version contains supplementary material available at 10.1186/s13293-024-00644-w.

## Highlights

XX DSD pigs have abnormal development of reproductive organs showing both male and female characteristics and lack of germ cells in the seminiferous tubules.

Whole-genome de novo sequencing was used to reveal the characteristics of genomic variants in XX DSD pigs.

The nucleotide changes of *IFITM1* (c.218T > C), *NOBOX* (c.1043 C > G), and a 70 bp deletion fragment of *WWOX* were the dominant variants in XX DSD pigs, and these three genes were significantly changed in the gonadal expression levels of the XX DSD pigs compared to those of normal females.

**Supplementary Information:**

The online version contains supplementary material available at 10.1186/s13293-024-00644-w.

## Introduction

Differences of sex development (DSD) is a group of congenital conditions that are defined by a discordance of the chromosomal, gonadal or phenotypic features of the internal and/or external genitalia [1]. DSD is a significant health concern among mammals, including pigs, goats, dogs, and even humans [2–5]. Based on the sex chromosome constitution, DSD can be classified into three categories: sex chromosome DSD, XX DSD, and XY DSD. In pigs, XX DSD (*SRY*-negative) is the most common type, which were also called intersexes or sex reversal in the past. Typical features of this condition include 38, XX karyotype, the intersex phenotype of genitalia, and the testis-like gonads or ovotestis [4]. XX DSD pigs are infertile, more susceptible to urogenital infections, and have reduced growth and survival rates. In addition, they exhibit a strong boar taint which diminishes carcass quality and may display aggressive behavior, ultimately compromising the economic returns of pig breeders [6]. The heritability estimates for XX DSD pigs are high, ranging from 0.72 to 0.81 [7, 8]. The incidence of this condition in various pig breeds ranges from 0.08 to 0.75%, but in certain inbred populations, it can be as high as 20%. XX DSD pigs seem to have a strain-dependent predisposition because, in most reported cases, the affected individuals belong to the Landrace pig or Large White pig or their hybrid [7, 9].

The molecular background of XX DSD (*SRY*-negative) has been extensively studied in humans. Multiple genetic variants have been linked to 46, XX testicular or ovotesticular DSD, including *RSPO1* [10, 11], *WNT4* [12], *NR5A1* [13, 14], *NRF2* [15, 16], *WT1* [17, 18], *SOX3* [19], *SOX9* [20, 21], and *SOX10* [22]. Despite progress in understanding the etiology of 46, XX testicular or ovotesticular DSD, the etiology of some cases is still unknown [23]. However, the causative variants for XX DSD have only been identified in goats among domestic mammals. In goats, the causative variant for the polled intersex syndrome is a complex structural variant resulting from a 10.1 kb fragment deletion and a duplicated 480 kb segment insertion, which affects the expression of *FOXL2* [7, 24]. The molecular background of XX DSD pigs remains incompletely understood. It had been suggested that XX DSD pigs are caused by an unknown autosomal recessive mutation [25, 26]. The *SOX9* was ever considered the primary candidate gene for investigating the molecular background of 38, XX DSD (*SRY*-negative). Rousseau et al. [8] conducted a genome-wide association analysis, and nine SNPs were identified in XX DSD pigs, with the rs81341093 (M1GA0024789) haplotype being the most significant. The authors also detected polymorphic loci in *SOX9* (10 SNPs and four indels) and the TESCO enhancer (14 SNPs and one indel), but none of these were co-segregated with the XX DSD phenotype. Brenig et al. [27] discovered an 18 bp deletion (Δ18, ss2137497745) in the 5’ UTR region of the *SOX9* gene and suggested that this deletion decreased *SOX9* transcriptional and translational activities. However, subsequent research by Stachowiak et al. [28] failed to find a correlation between Δ18 and the XX DSD phenotype. Instead, these researchers revealed a copy number variation (CNV) region downstream of *SOX9* (~ 500 kb) in four XX DSD pigs using fluorescence in situ hybridization (FISH), whic*h* was also observed in another two XX DSD pigs [29], this means it is likely a molecular marker for the XX DSD pig, but further validation from a larger cohort is needed.

Therefore, it is imperative to identify the causative gene responsible for XX DSD pigs and subsequently develop precise molecular diagnostic techniques based on this gene to identify and manage carriers. This will optimize the germplasm resources of breeding pigs. Furthermore, studying XX DSD pigs could provide crucial insights into the etiology of human DSD and enhance our comprehension of mammalian sex differentiation. Previous studies suggested that whole-genome sequencing and de novo assembly can detect more SNPs and SVs while improving the accuracy of variant detection [30, 31]. In this study, we performed whole-genome de novo sequencing on normal female pigs and XX DSD pig using 10× Genomics sequencing technology. Candidate variants obtained from genomic variant analysis were validated in a larger group of XX DSD pigs. These variants are expected to be molecular breeding markers for XX DSD pigs and provided a theoretical basis for elucidating the molecular background of XX DSD pigs.

## Materials and methods

### Animals

After euthanasia, the reproductive organs of XX DSD, normal female, and normal male pigs were dissected and compared. We collected partial gonadal tissues from 3 XX DSD pigs, 3 normal female pigs, and 3 normal male pigs. These tissues were rapidly frozen in liquid nitrogen and stored at -80 °C for further experiments. Additionally, some gonadal tissues were fixed in 10% neutral buffered formaldehyde, embedded in paraffin, and sectioned at a thickness of 5 μm for histological analysis. The sections were stained with hematoxylin and eosin (HE). Primers specific for internal reference gene *GAPDH* were designed using Primer 5 (Table S1). The previously published study provided us with *SRY*-specific primers [32]. The duplex PCR reaction was performed using the Multiplex PCR Kit (Vazyme Biotech, Nanjing, China, PM101) according to the manufacturer’s instructions. After PCR detection (lack of *SRY* gene), we selected one normal female pig (NF) and two XX DSD pigs (D1 and D2) at one month of age for whole-genome sequencing using genomic DNA.

We randomly collected 32 normal female pigs and 32 XX DSD pigs (Fig. S1) for the validation experiments of candidate genomic variant loci and immediately froze their ear tissues in liquid nitrogen. Subsequently, we stored the samples in a -80 °C freezer until DNA isolation.

### 10× Genomics library construction and sequencing

Using 10× Genomics sequencing technology, we perform WGS and de novo assembly on three Yorkshire pigs (NF, D1 and D2). To perform 10× Genomics sequencing, high-molecular-weight (HMW) genomic DNA was extracted from blood, indexed and barcoded according to the Genome Reagent Kit Protocol (10× Genomics). Briefly, in the 10× Genomics Chromium microfluidic Genome Chip, approximately 1 ng of HMW DNA was combined with master mix and partitioning oil to create Gel Bead-in-Emulsions (GEMs). During the GEM incubation, Read 1 sequence, 10× Genomic barcode, and random primer sequence were added. P5 and P7 primers, Read 2, and sample index were added during library construction after end repair, A-tailing, and adaptor ligation and amplification. Subsequently, the library was sequenced using 150PE mode on the Illumina HiSeq 2500 platform.

### Genome assembly and evaluation

Raw reads were first processed using Trimmomatic version 0.38 for quality control to obtain clean reads [33]. Clean reads were de novo assembled with SOAP denovo (version v2.04) software [34], followed by gap filling. The assembly procedures comprised the following steps: splitting up the reads into k-mers, constructing a de Bruijn graph, producing contig sequences, realigning reads to contig sequences to construct scaffolds, and using GapCloser (version 1.12) to fill gaps within these scaffolds. The integrity of the assembled genome was evaluated using BUSCO version 5.2.2 based on the single-copy homologous gene set in OrthoDB (http://cegg.unige.ch/orthodb).

### SNPs mining and validation

To identify SNPs, we aligned the assembled sequence to the reference genome (*Sus scrofa* 11.1) with LASTZ software (http://www.bx.psu.edu/~rsharris/lastz/). To exclude assembly errors, validation of SNPs was assisted by aligning reads to the reference and assembled genomes, respectively. Variant annotation was performed with Annovar (http://www.openbioinformatics.org/annovar/). SNPs were screened according to the autosomal recessive inheritance pattern with the following criteria: (A) Screening for homozygous nonsynonymous mutations only present in XX DSD pigs; (B) Screening for homozygous nonsynonymous mutations only present in normal female pig; (C) Screening for nonsynonymous mutations that are homozygous in XX DSD pigs and heterozygous in normal female pig; (D) Screening for heterozygous nonsynonymous mutations only present in normal female pig. The genes harboring the screened SNPs were subjected to Gene Ontology (GO) functional annotation and Kyoto Encyclopedia of Genes and Genomes (KEGG) pathway analysis using the clusterProfiler R package. We screened candidate SNPs based on gene information from public databases and from the literature.

For validation, we designed specific primers against the candidate SNPs and performed PCR amplification (Table S2). The PCR amplification products were then sent to Sangon Biotech (Shanghai, China) for Sanger sequencing. For gene expression analysis, total RNA was extracted from gonadal tissue using the FastPure Cell/Tissue Total RNA Isolation Kit V2 (RC112-01, Vazyme, Nanjing, China). RNA was isolated from a total of nine animals, divided into three groups: normal females (*n* = 3), XX DSDs (*n* = 3), and normal males (*n* = 3). cDNA was generated using the HiScript III All-in-one RT SuperMix Perfect for qPCR (R333-01, Vazyme, Nanjing, China). For quantitative real-time PCR (qRT-PCR), cDNA, primers, and the ChamQ Universal SYBR qPCR Master Mix (Q711, Vazyme, Nanjing, China) were mixed following the manufacturer’s instructions. Each qRT-PCR reaction was performed in triplicate for each individual. The relative expression levels of target genes were calculated using the delta-delta Ct method in Microsoft Excel. Primer sequences are provided in Table S3.

### SVs mining and validation

SVs were identified using LASTZ software, following the same alignment method used for SNPs. SVs were screened using the following two steps: (A) Specific SVs unique to D2 when compared to NF were screened; (B) The SVs screened in the first step were further compared with those of D1, and only SVs with the same variant gene positions and types were selected. Then, we performed GO and KEGG enrichment analysis of the genes harboring SVs to screen for candidate SVs. The candidate SVs were validated by CNVplex assays according to the manufacturer’s instructions (GENESKY, China) [35]. The Actin beta gene (ACTB), Collagen type X alpha 1 chain gene (COL10A1), and glucagon gene (GCG) were served as internal reference controls for CNVplex assays. All specific primers of each candidate SVs were listed in Table S4. Briefly, the ligation reaction was performed in a 20 µl volume for each of the 32 control females and 32 XX DSD pigs, containing 10× ligase buffer (2 µl), ligase (0.5 µl), probe mix (1 µl), ddH_2_O (7.5 µl), and 120 ~ 200 ng genomic DNA. The program of ligation reaction was as follows: 4 cycles of 94 ºC for 1 min and 60 °C for 4 h; 94 ºC for 2 min; hold at 72 °C until 20 µl of 2 × Stop Buffer was added in. Then the ligation products were subjected to a multiplex fluorescence PCR amplification. The PCR reaction was performed in 20 µl for each sample, containing 2× PCR Master Mix (10 µl), probe mix (1 µl), ligation product (1 µl), and ddH_2_O (8 µl). The PCR program was as follows: 95 °C for 2 min; 30 cycles of 94 °C for 20 s, 57 °C for 40 s and 72 °C for 1.5 min; 60 °C for 60 min; 4 °C forever. PCR products were diluted 15-fold before being loaded on the ABI3730XL sequencer. Raw data were analyzed by GeneMapper 5.0 (Applied Biosystems, USA). The relative copy number of candidate SVs was determined by the ratio of the copy number of the target segment to the average copy number of the three reference genes as previously described [35].

In order to confirm the 70 bp deletion of the *WWOX* gene, we selected a pair of specific PCR primers (Forward: ATCCTCGCAGGACACAGGAG, Reverse: TGTGTAGCGGCCTCCAGAAG) to cover the 70 bp deletion for Sanger sequencing. The annealing temperature was 60 ℃ and the PCR products were separated into wild-type (298 bp) and deletion (228 bp) bands on a 2% agarose gel. The gonad tissue sections underwent deparaffinization, rehydration, and sequential heat-mediated antigen retrieval using EDTA buffer (pH = 9.0). Subsequently, the sections were blocked with 3% bovine serum albumin (BSA) in TBST for 30 min at room temperature. After blocking, primary antibodies were incubated overnight at 4 °C. The primary antibody used was rabbit anti-WWOX (15299-1-AP; Proteintech, Wuhan, China, 1:300). The next day, the sections were washed and incubated with the corresponding secondary antibodies for 50 min at room temperature. The secondary antibody utilized was goat anti-rabbit IgG labeled with Alexa Fluor 488 (GB25303; Servicebio, Wuhan, China, 1:400). Following incubation with the secondary antibodies, DAPI solution was applied to stain the nuclei. Finally, immunofluorescence images were captured using Fluorescent Microscopy (NIKON ECLIPSE C1, NIKON).

## Results

### Characteristics of XX DSD pigs and de novo genome assembly

**Fig. 1:**
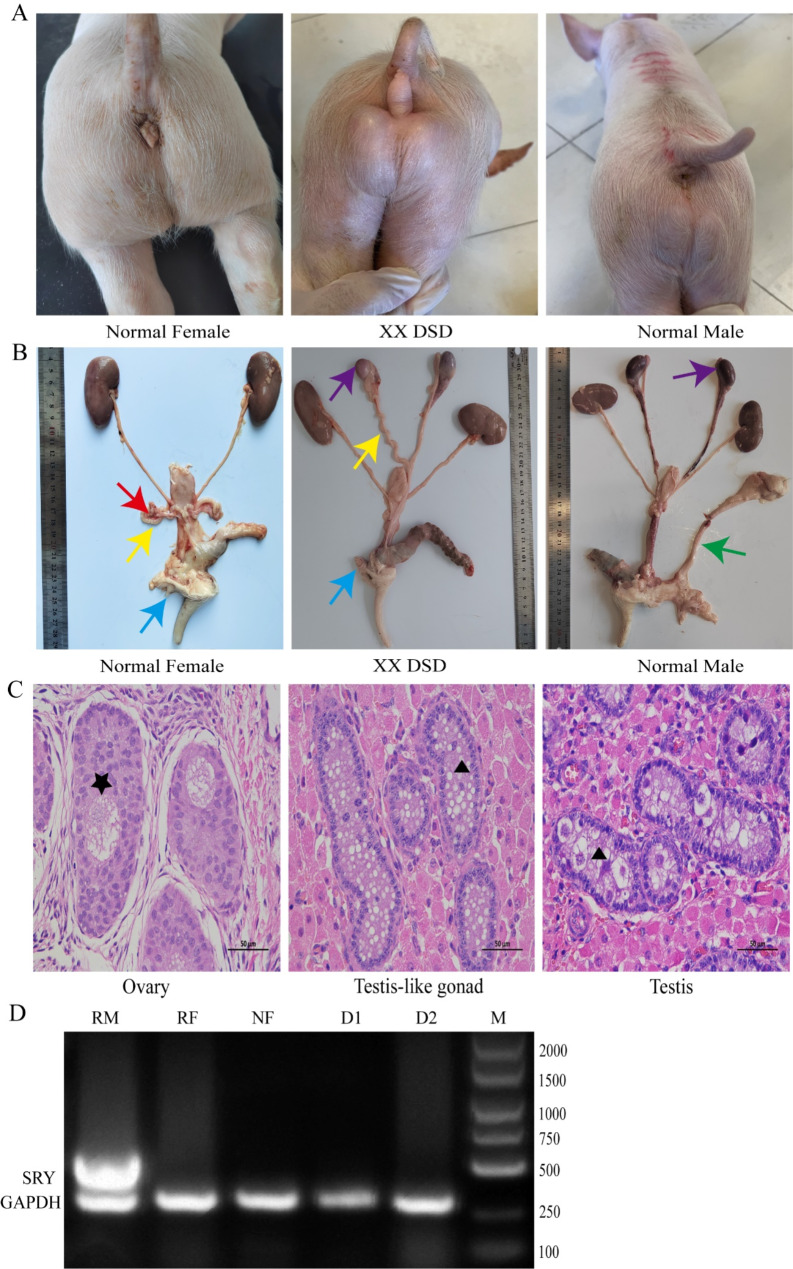
Characteristics of 1-month-old XX DSD pigs. (**A**) External genitalia characteristics of XX DSD pig. (**B**) Internal genitalia characteristics of XX DSD pig. Red arrow stands for ovary; yellow arrows stand for uterine horns; blue arrows stand for the vulva; purple arrows stand for testes; green arrows stand for the penis. (**C**) Histological analysis of the gonads in XX DSD pigs. HE staining was used to analyse the histological characteristics of the ovaries of normal female pigs, the testes of normal male pigs and the testis-like gonads of XX DSD pigs. Black stars indicate primary follicles; black triangles indicate the lumen of the seminiferous tubules. (**D**) Duplex PCR for *SRY* gene detection using *GAPDH* as an internal reference gene; M stands for the marker, RM stands for reference male, RF stands for reference female, NF stands for normal female pig, and D1 and D2 stand for XX DSD pigs

XX DSD pigs display both boar and sow reproductive organ characteristics. Visual examination of XX DSD pigs revealed two scrotal masses resembling testes and an enlarged, malformed vulva (Fig. 1A). Anatomical examination of XX DSD pigs revealed the presence of Miillerian and Wolffian ducts derivatives in their internal genitalia. They had two oval-shaped gonads resembling testes and curly uterine horn (Fig. 1B). HE staining revealed the presence of primary oocytes in normal ovaries and seminiferous tubules in normal testes. Additionally, seminiferous tubules were observed in the testis-like gonads of XX DSD pigs. However, these XX DSD pigs had a lack of germ cells and a significant presence of empty vacuoles in the ducts (Fig. 1C). Molecular biology tests confirmed the absence of *SRY* gene in XX DSD pigs (Fig. 1D).

To identify genomic variants in XX DSD pigs, we constructed de novo genome assemblies using 10× Genomics linked-read technology and evaluated the assembly results. Table 1 shows the statistics of the de novo genome assemblies of each of the three individuals. The clean sequencing data for each individual exceeded 160 Gb, with an estimated average sequencing depth of > 60× based on pig genome sizes. The clean data of normal female (NF) was assembled into a genome of 2.46 Gb with a contig N50 length of 92.3 kb and scaffold N50 length of 5.46 Mb. The XX DSD pigs (D1 and D2) genomes were assembled with sizes of 2.47 Gb and 2.46 Gb, respectively. The contig N50 lengths for D1 and D2 were 82.2 kb bp and 93.3 kb bp, while their scaffold N50 lengths were 7.83 Mb and 7.30 Mb, respectively. Next, we evaluated assembly quality using BUSCO, with each sample exhibiting > 90% complete single-copy BUSCOs (Table 1). These results indicate high accuracy and completeness of the genome assembly. We used these genomes for the subsequent analysis of genomic variant.

**Table 1:** Summary of genome assembly results and evaluations

	NF	D1	D2
Assembly statistics			
Clean data (Gb)	174	177	166
contig N50 (bp)	92,341	82,287	93,360
scaffold N50 (bp)	5,462,448	7,828,145	7,398,794
Scaffold (Gb)	2.46 Gb	2.47 Gb	2.46 Gb
Contig number	71,561	83,579	71,417
Scaffold number	26,640	32,805	26,986
BUSCOs evaluation			
Complete Single-Copy BUSCOs (%)	92	90	91
Complete Duplicated BUSCOs (%)	3.00	2.60	2.40
Fragmented BUSCOs (%)	3.00	4.30	3.20
Missing BUSCOs (%)	4.50	4.80	4.80
Total BUSCO groups	843	843	843

### SNPs analysis

**Fig. 2:**
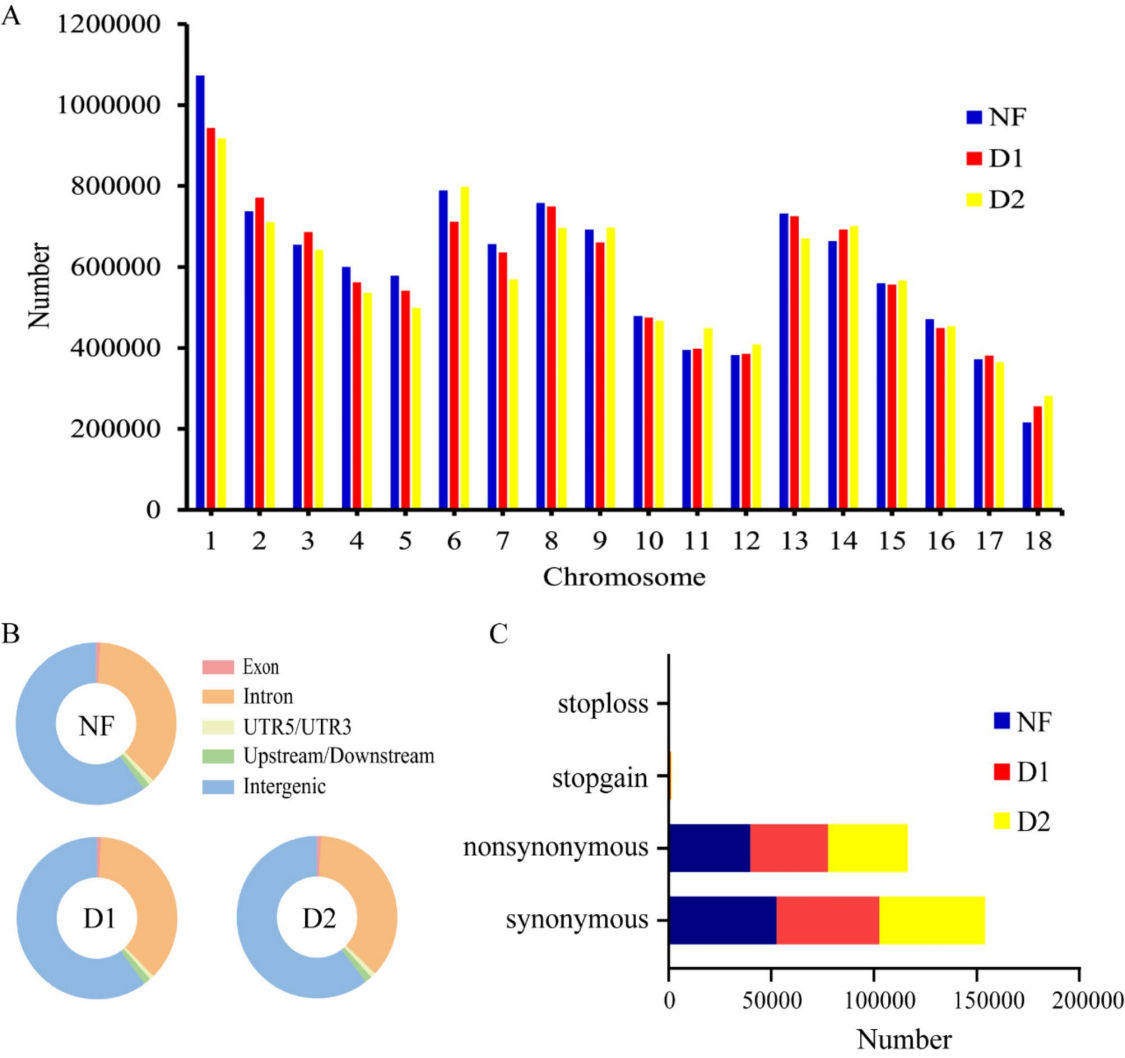
Distribution characteristics of SNPs on chromosomes in three Yorkshire pigs (NF, D1, and D2). (**A**) The number of SNP distribution on chromosomes; (**B**) Distribution of SNPs on chromosomes. (**C**) Number of SNPs with different mutation types in the exon region

**Fig. 3:**
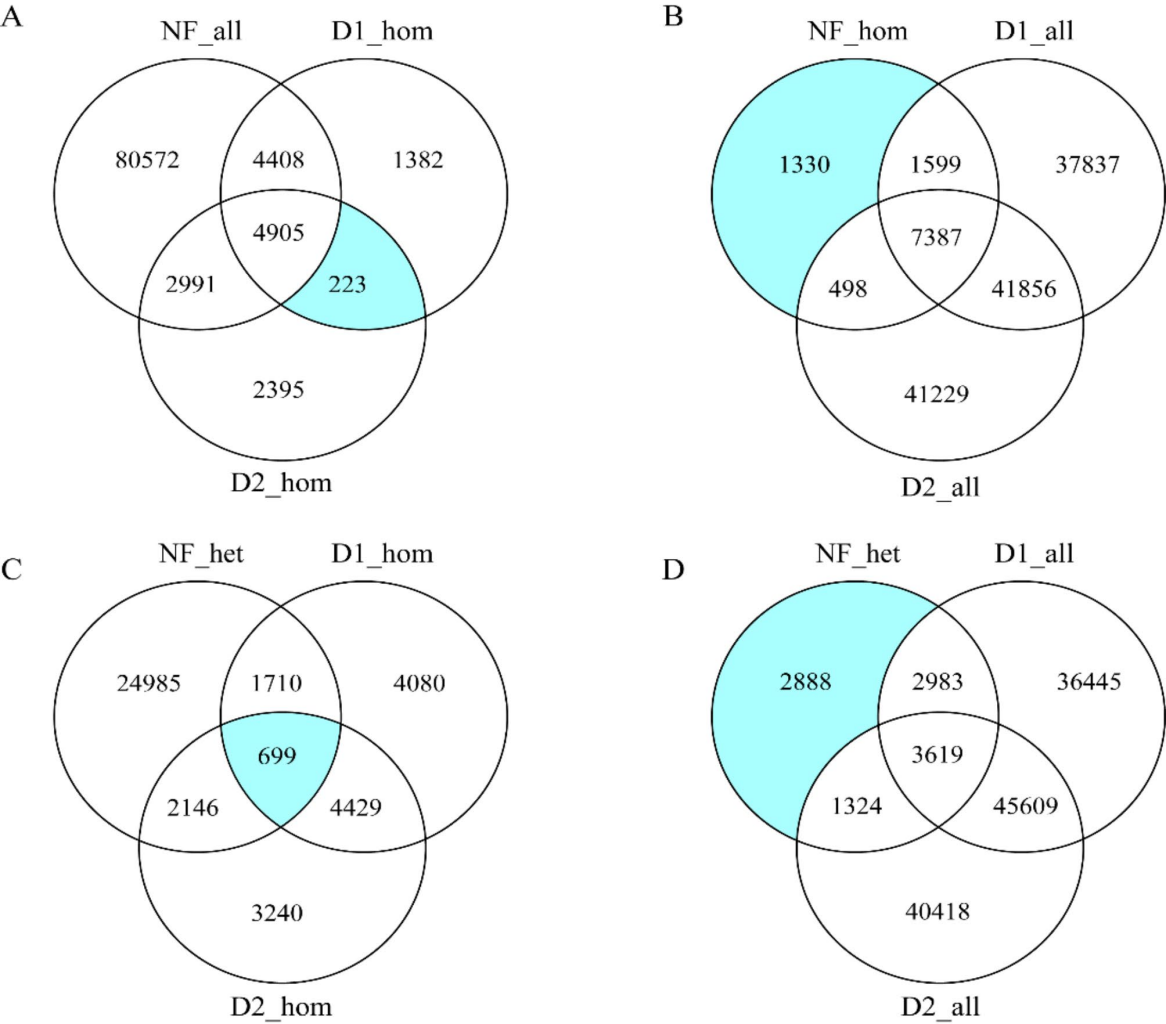
SNPs screen according to recessive inheritance pattern. (**A**) Screening for homozygous nonsynonymous mutations only present in XX-DSD pigs; NF_all stands for all non-synonymous SNPs in normal female pigs; D1_hom and D2_hom stand for homozygous SNPs with non-synonymous mutations in D1 and D2, respectively (same as below). (**B**) Screening for homozygous nonsynonymous mutations only present in normal females. NF_hom stands for homozygous SNPs with non-synonymous mutations in normal female pigs; D1_all and D2_all stand for all non-synonymous SNPs in D1 and D2, respectively (the same as below). (**C**) Screening for nonsynonymous mutations that are homozygous in XX-DSD pigs and heterozygous in normal female. NF_het stands for heterozygous SNPs with non-synonymous mutations in normal female pigs (the same as below). (**D**) Screening for heterozygous nonsynonymous mutations only present in normal female pigs

The genomes of NF, D1, and D2 were aligned with the reference genome to identify 11,549,414, 11,331,915, and 1,203,772 SNPs, respectively. Chromosomal distribution statistics showed similar patterns of SNPs among the three individuals (Fig. 2A). Analysis of SNP mutation types revealed that more than 95% of SNPs were located in intronic and intergenic regions (Fig. 2B). Among the SNPs located in the exonic regions, the majority were synonymous mutations (56.53%), followed by nonsynonymous mutations (42.76%) (Fig. 2C). According to the literature, we screened SNPs for nonsynonymous mutations using a recessive inheritance pattern, and a total of 5140 SNPs were screened (Fig. 3). It is worth mentioning that none of these SNPs were found to be located in the *SOX9* gene. Next, the genes harboring these SNPs were analyzed using Gene Ontology (GO) and Kyoto Encyclopedia of Genes and Genomes (KEGG). These genes were enriched for the Steroid hormone biosynthesis signaling pathway and GO terms related to enzyme activity (Fig. S2). Candidate SNPs were carefully screened using GO and KEGG analyses, gene information from public databases, and gene functions and diseases associated with sexual developmental processes in the literature. After that, a total of 27 SNPs derived from 11 genes were ultimately identified as candidate loci, and their details are presented in Table 2.

**Table 2:** Information on the 27 candidate SNPs used for the XX DSD pig cohort validation

Genes	Genomic Position	Exon	SNP Site	Gene function/disorders associated
*IFITM1*	2:152691	exon1	c.8 G > A	*IFITM1* is involved in migration and development of primordial germ cells, and serves as a downstream target of the WNT/β-catenin signalling pathway [41, 42].
	2:152822	exon1	c.139 A > C	
	2:125206	exon2	c.227 T > C	
	2:153536	exon2	c.218 T > C	
	2:153638	exon2	c.320 C > T	
	2:153677	exon2	c.359G > A	
	2:125327	exon2	c.348T > G	
	2:125347	exon2	c.368G > A	
*LHR*	3:91964504	exon1	c.26G > A	Mutations in this gene cause Leydig cell hypoplasia with male pseudohermaphroditism (Leydig cell hypoplasia type I) [69].
	3:91964548	exon1	c.70 A > G	
*ZFPM2*	4:31610023	exon8	c.1333T > C	Mutations in this gene cause 46, XY Sex Reversal 9 [70]
*HSD17B6*	5:22105242	exon2	c.275 C > T	Enzyme involved in synthesis of steroid hormones [71].
	5:22105118	exon2	c.151G > C	
*WNT4*	6:80112670	exon5	c.861 C > A	WNT4 plays a key role in female reproductive structure development; mutations in this gene cause 46, XX DSD (Serkal Syndrome) [12].
*BMP8B*	6:95573546	exon2	c.487G > A	Defective primordial germ cell formation in *BMP8B* knockout mice [72]; diseases associated with *BMP8B* include Primary ovarian insufficiency (POI) [73].
	6:95573622	exon4	c.767 C > T	
	6:95573631	exon4	c.691G > A	
	6:95584620	exon4	c.682G > A	
*POU5F1*	7:23570432	exon1	c.169G > A	*POU5F1* plays a key role in embryonic development; diseases associated with *POU5F1* include Embryonal Carcinoma and Germinoma [74].
*AMHR2*	8:18648724	exon10	c.1472G > A	Mutations in this gene cause DSD in human [75, 76].
*NOBOX*	9:113350107	exon2	c.131 G > A	Diseases associated with *NOBOX* include Premature Ovarian Failure 5; *NOBOX* deficiency disrupts early folliculogenesis and oocyte-specific gene expression [43,46].
	9:113351498	exon4	c.781 G > T	
	9:113352885	exon5	c.1043 C > G	
	9:113352945	exon5	c.1103 C > A	
*LHX9*	10:20759802	exon5	c.1114 G > A	*LHX9* is associated with gonadal development; mutations in this gene related to 46, XY DSD [77, 78].
	10:20759803	exon5	c.1115 T > C	
*CFTR*	28,783,462	exon1	c.80 C > T	Mutations in *CFTR* are associated with oligozoospermia, epididymal obstruction, congenital bilateral absence of vas deferens (CBAVD) and idiopathic ejaculatory duct obstruction (EDO) [79].

**Fig. 4:**
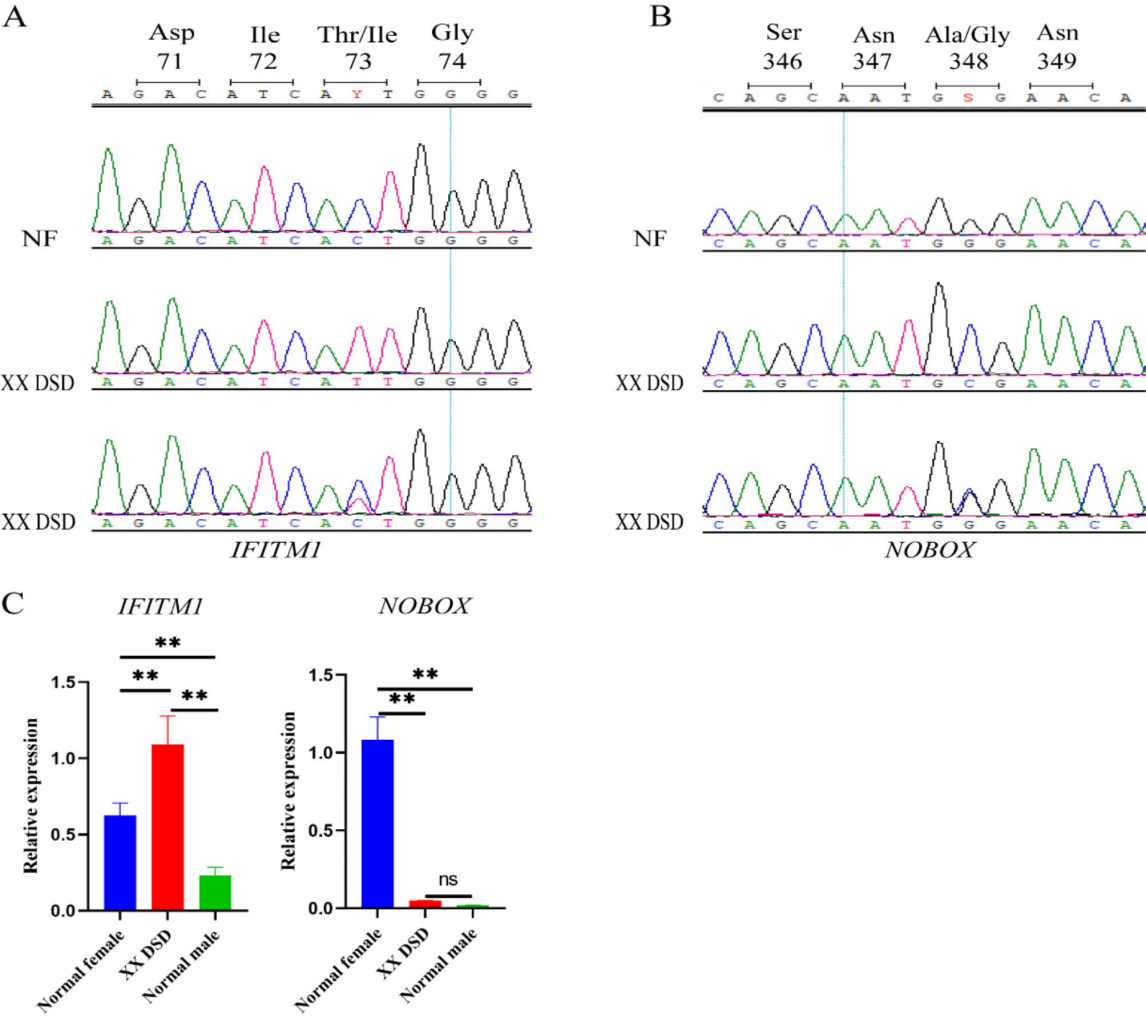
Comparison of SNPs and relative mRNA expression of candidate genes. (**A**) The SNP (c.218T > C) in the *IFITM1*. (**B**) The SNP (c.1043 C > G) in the *NOBOX*. (**C**) The relative expression of *IFITM1* and *NOBOX* mRNA was detected by qRT-PCR (*n* = 3 for each group; ** *P* < 0.01, ns *P* > 0.05)

The candidate SNPs were validated on larger cohorts (32 normal female pigs and 32 XX DSD pigs) by Sanger sequencing to further identify SNPs with genotype frequencies that differed between normal female pigs and XX DSD pigs. Validation results revealed the presence of heterozygous mutations in the XX DSD pig cohort despite our screening for SNP loci in a recessive inheritance pattern (Table S5). It is noteworthy that the SNP (c.218T > C) in the *IFITM1* and the SNP (c.1043 C > G) in the *NOBOX* were not evenly distributed between the affected and control animals (Table 3). The TT and TC genotypes of the SNP in the *IFITM1* and the CC and CG genotypes of the SNP in the *NOBOX* were exclusively found in XX DSD pigs. Here, the SNP of *IFITM1* gene changed the polar amino acid threonine (Thr) to the nonpolar amino acid isoleucine (Ile). In addition, the hydrophobic alanine (Ala) changed to hydrophilic glycine (Gly) due to the SNP of *NOBOX* (Fig. 4AB). To further understand the expression levels of *IFITM1* and *NOBOX* genes in gonadal tissues, we used qRT-PCR to determine their relative expression levels. A significant difference existed in the expression levels of these two genes between affected and control gonads. The expression of *IFITM1* mRNA in the gonads of XX DSD pigs was significantly higher than that in normal female and normal male pigs (Fig. 4C). Additionally, *NOBOX* mRNA expression in the gonads of XX DSD pigs was significantly lower compared to normal female pigs. Notably, *NOBOX* mRNA was scarcely expressed in the gonads of XX DSD pigs and normal male pigs. For the other genes suggested by WGS in XX DSD, no significant associations were found in the larger cohort validation because of the similar distribution of SNP variants in normal female pigs and XX DSD pigs (Table S5).

**Table 3:** Genotype and SNP variant distribution of IFITM1 and NOBOX obtained by Sanger sequencing

Genes	Site	XX DSD pigs (*n* = 32)		Normal female pigs (*n* = 32)	
		Genotype frequencies	Allele frequencies	Genotype frequencies	Allele frequencies
*IFITM1*	c.218 T > C	TT = 0.875	T = 0.938	TT = 0	T = 0
		TC = 0.125	C = 0.062	TC = 0	C = 1
		CC = 0		CC = 1	
*NOBOX*	c.1043 C > G	CC = 0.813	C = 0.906	CC = 0	C = 0
		CG = 0.187	G = 0.094	CG = 0	G = 1
		GG = 0		GG = 1	

### SV analysis

**Fig. 5:**
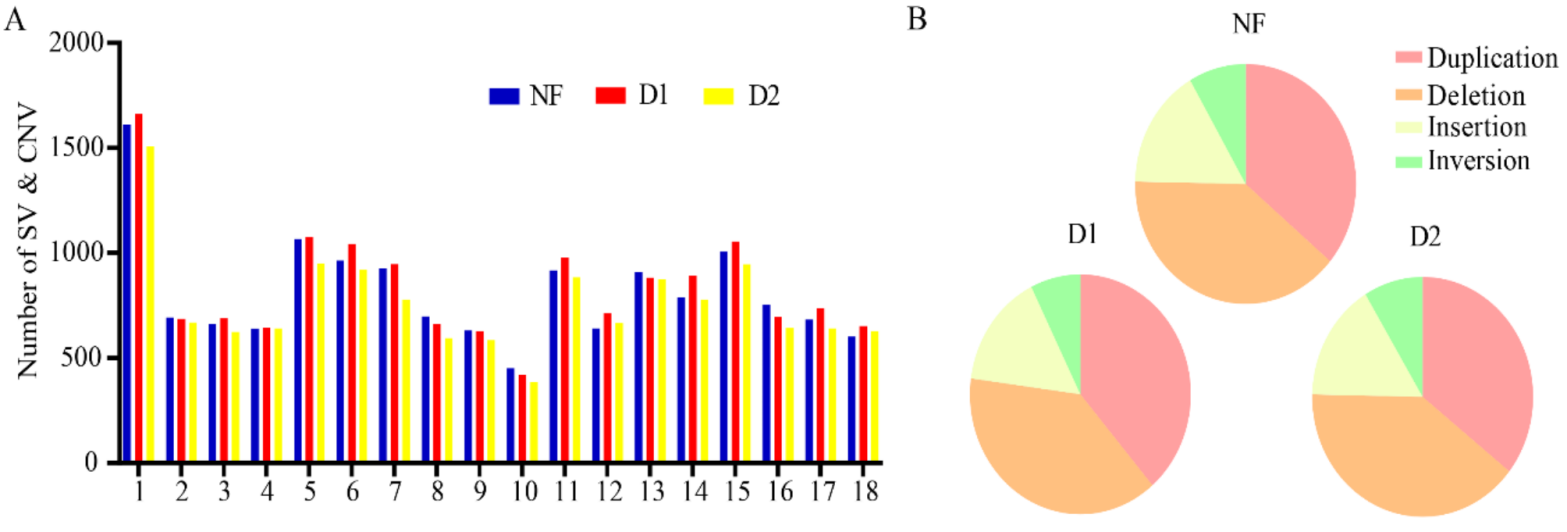
Characteristics analysis of SVs in three Yorkshire pigs (NF, D1, and D2). (**A**) Distributions of SVs on chromosomes. (**B**) The proportion of different SVs

The assembled genomes of NF, D1, and D2 identified 10,756, 11,364, and 10,223 SVs, respectively. Statistical analysis indicated that the distribution pattern of SVs on chromosomes was similar to that of SNPs, with deletions and duplications being the most common SV types and inversions the least common (Fig. 5). NF served as the control and was compared with D1 and D2 to identify SVs with the same location and variant type. GO and KEGG analyses were performed on the 1,474 genes where these SVs were located (Fig. S3). The GO analysis showed that genes harboring SVs were mainly enriched in signaling and signaling receptor activities. The KEGG analysis results showed that these genes were enriched in steroid hormone biosynthesis, cAMP signaling pathway, WNT signaling pathway, and MAPK signaling pathway. Similar to the screening method for candidate SNPs, 14 candidate SVs were finally screened for the further study (Table 4).

**Table 4:** Information on the 14 candidate SVs used for the XX DSD pig cohort validation

Type	Genes	Genomic Position	Size	Gene function/disorders associated
Inversion	*TBP*	1:1-5433	5,433 bp	This gene is associated with spermatogenesis [80].
Deletion	PITX1*	2:137200501–137,209,200	8,700 bp	*PITX1* regulates the synthesis of reproductive hormones and the expression of related genes, including *LHβ*, *FSHβ*, and *GNRHR* [81, 82].
Deletion	PDGFA/PRKAR1B/DNAAF5*	3:231801–479,100	247,300 bp	*PDGFA* is associated with Leydig cell development and testosterone synthesis [83].
Duplication	*SRD5A2*	3:107856801–107,907,700	50,900 bp	Variants in this gene cause 46, XY DSD [3].
Duplication	SHC1*	4:94822101–94,826,100	4,000 bp	SHC1 is involved in oocyte maturation [50].
Duplication	*RORC*	4:97372801–97,377,100	4,300 bp	Key regulators involved in steroid metabolism [84].
Duplication	*WNT2B*	4:107914901–107,923,000	8,100 bp	This gene functions in the Wnt/β-catenin signalling pathway and regulates the proliferation of ovarian granulosa cells [85,86].
Inversion	*SYCE3*	5:185339–186,259	921 bp	Syce3 regulate testosterone and dihydrotestosterone synthesis via steroidogenic pathways in Sertoli and Leydig cells [87].
Deletion	WWOX*	6:9669113–9,669,182	70 bp	*WWOX* plays a role in sex hormone synthesis and gonadal development; associated with human and dog DSD [2, 66,68].
Deletion	*CCDC85C*	120,582,001–120,616,000	34,000 bp	This gene is involved in the beta-collagen signalling pathway [88].
Inversion	*NXPH1*	9:79527380–79,534,749	7,370 bp	This gene is associated with ovarian development [89].
Deletion	*EGFR*	9:139446401–139,499,300	52,900 bp	This gene is located in the EGFR Signaling Pathway and is associated with testicular development [90, 91].
Duplication	SOX9*	12:8562001–8,572,600	10,600 bp	*SOX9* plays an important role in male sex determination and differentiation; variants in this gene cause DSD in mammals [3, 20].
Deletion	WNT6*	15:120911501–120,914,400	2,900 bp	*WNT6* activates the WNT/β-catenin signalling pathway [92]; involved in spermatogenesis [54].

Note: * represent the locus which occured copy number variation in the XX DSD pig cohort. *SOX9* stands for CNV in the region downstream of the *SOX9* gene

**Fig. 6:**
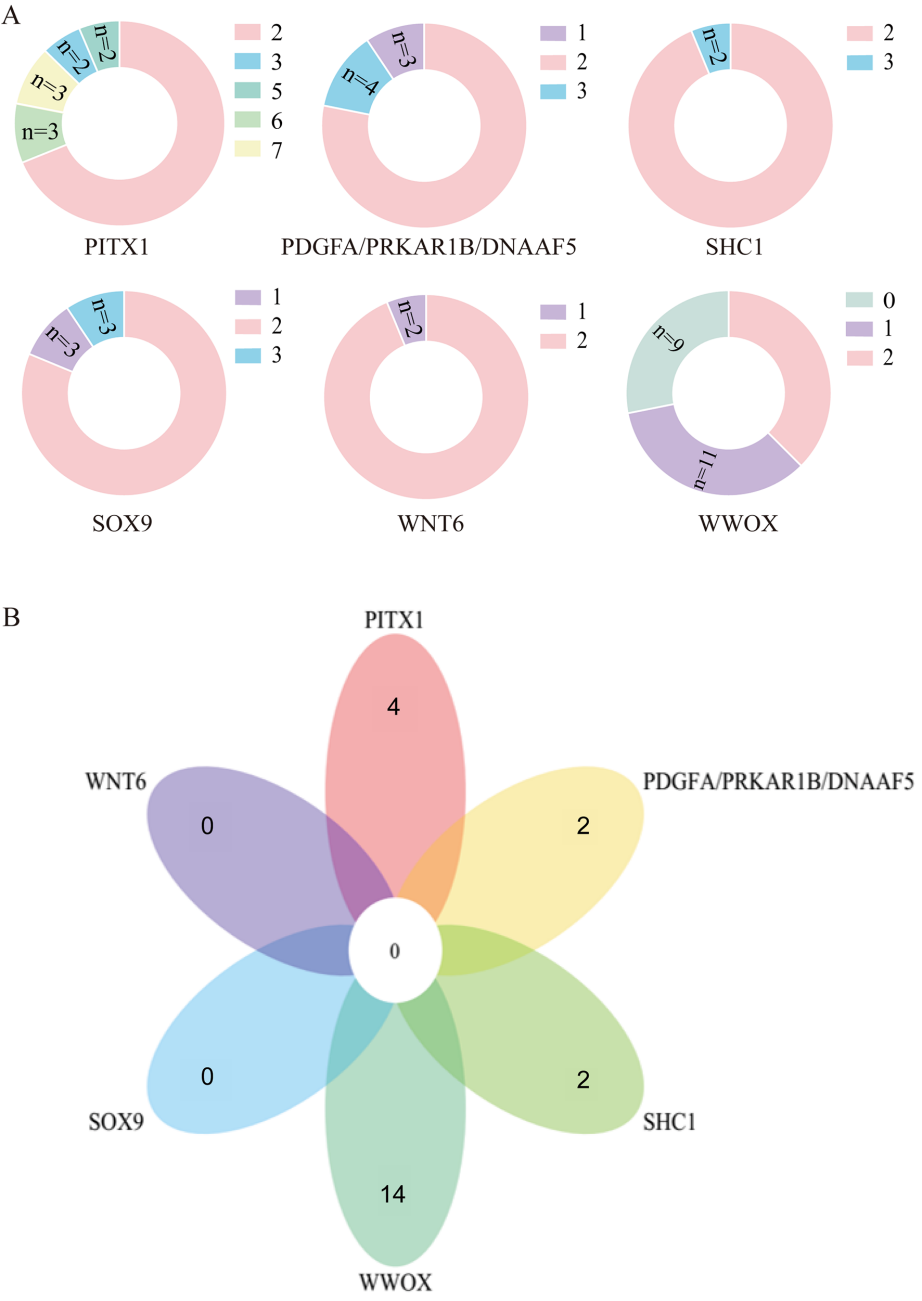
Validate candidate SVs in the XX DSD pig cohort. (**A**) The six SVs displayed altered copy numbers in the XX DSD pig cohort. (**B**) A total of 22 individuals with only one SV were detected in the XX DSD pig cohort. *SOX9* stands for CNV in the region upstream of the *SOX9* gene

We used CNVplex assay to validate candidate SVs in a cohort containing 32 normal female pigs and 32 XX DSD pigs. The validation results demonstrated that six SVs displayed altered copy numbers in the XX DSD pig cohort (Fig. 6A). However, the copy numbers of these six SVs remained unchanged in the vast majority of normal female pigs (Table S6). Specifically, 62.5% of XX DSD pigs had a 70 bp deletion in intron 5 of the *WWOX* gene, either with 0 or 1 copy of the deletion, while 18.75% of XX DSD pigs exhibited CNVs in the downstream region of the *SOX9* gene (~ 70 kb). Additionally, 21.88% of XX DSD pigs showed copy number increase or decrease on the SV of chromosome 3 (231801–479100 bp), covering the genes *PDGFA*, *PRKAR1B*, and *DNAAF5*. Interestingly, in contrast to the WGS results, 31.25% of XX DSD pigs showed an increase in the copy number of the pituitary homeobox paired homeodomain transcription factor 1 gene (*PITX1*). Among the XX DSD pig cohort, 22 individuals had only one SV, while another 10 individuals had two or more SVs. (Fig. 6B). Specifically, 14 of these individuals presented just the *WWOX* deletion, while four individuals exclusively had the *PITX1* gene variant. Moreover, two individuals harbored solely the CNV of Src homology 2 domain containing transforming protein l (*SHC1*), and another two individuals possessed only the SV of chromosome 3 (located at 231801–479100 bp). However, XX DSD pigs with *WNT6* or the upstream region of the *SOX9* gene variants usually carried additional variants.

**Fig. 7:**
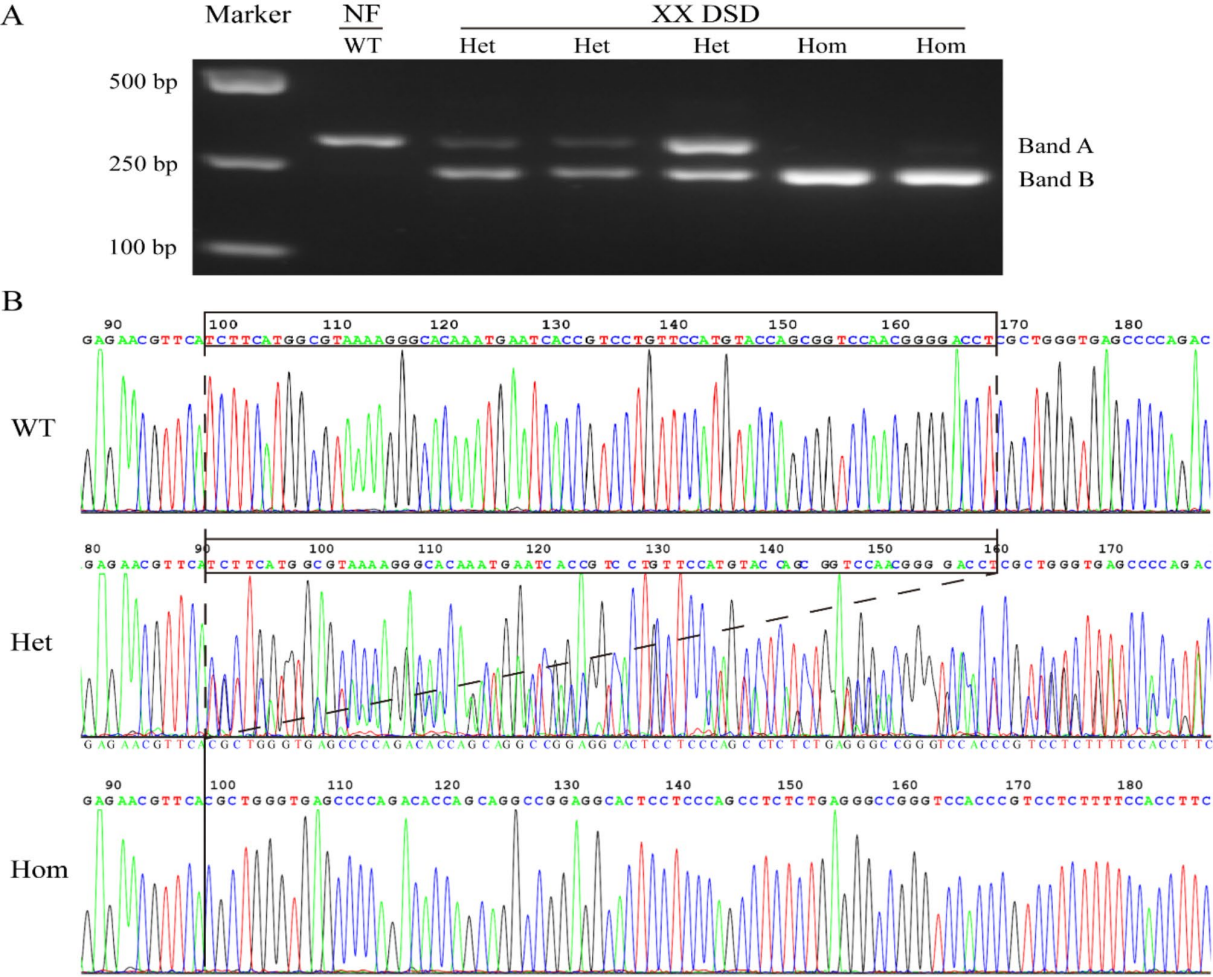
PCR and Sanger sequencing validated the 70 bp deletion of the *WWOX* gene. (**A**) Gel electrophoresis of PCR products derived from normal female pig exclusively displayed wild-type bands (referred to as band A, 298 bp). Conversely, in XX DSD pigs, discernible deletion bands surfaced, encompassing homozygous (referred to as band B, 228 bp) as well as heterozygous (228 bp and 298 bp) deletions. (**B**) The sanger sequencing results showed a 70 bp deletion between the wild type and homozygous type. The sequencing results of the heterozygous bands were manually corrected to show two strands with a 70 bp difference. Black boxes indicate the 70 bp deletion. WT indicates wild-type, Het indicates heterozygous, and Hom indicates homozygous

**Fig. 8:**
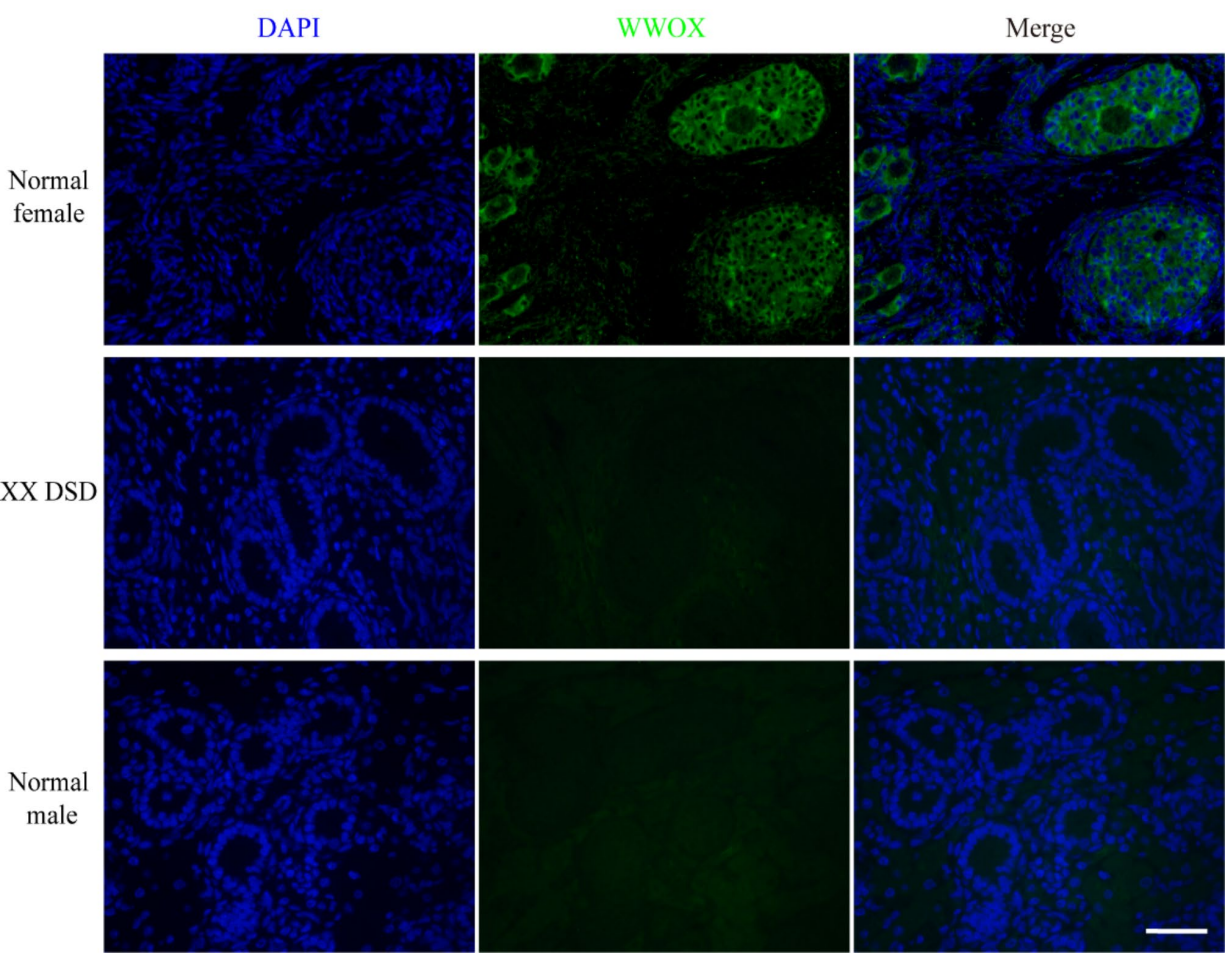
Comparison of WWOX immunofluorescence staining in gonads of XX DSD, normal female and normal male pigs. Bar = 50 μm

We futher employed PCR and Sanger sequencing techniques to validate the 70 bp deletion of the *WWOX* gene. The PCR product from normal female pigs showed a single band (band A, wild type). However, in XX DSD pigs, either one band (band B, homozygous deletion) or two bands (band A and band B, heterozygous deletion) were discerned (Fig. 7A). The outcomes obtained through Sanger sequencing exhibited a disparity of 70 base pairs between the sizes of band A and band B (Fig. 7B). This discrepant size firmly substantiates the presence of the *WWOX* gene deletion in XX DSD pigs. We proceeded to examine the expression of WWOX in gonadal tissues using the immunofluorescence staining method. The results revealed high expression of WWOX in the cytoplasm of ovarian follicular cells in normal female pigs. However, in the gonadal tissues of XX DSD pigs and normal male pigs, the expression of WWOX was almost imperceptible (Fig. 8). This finding implies that the *WWOX* gene plays a pivotal role in ovarian development, and the low expression of *WWOX* in XX DSD pigs is likely related to a 70 bp deletion in intron 5 of the *WWOX* gene.

## Discussion

Sex development relies on the sex-specific action of gene networks to differentiate the bipotential gonads of the growing fetus into testis or ovaries. DSD arises from congenital alterations during any of these processes; thus, elucidating the molecular background of DSD is a very challenging task [3]. Whole-genome sequencing is a comprehensive method for detecting disease-related genetic variants at the whole-genome level, including SNPs and SVs. It has been recommended for diagnostic studies of human DSD [36]. The second-generation sequencing lacks sensitivity and accuracy for detecting SV due to its short read length. The linked read sequencing library preparation platform by 10× Genomics produces barcoded sequencing libraries, which are subsequently sequenced using the Illumina short read sequencing technology. Large DNA fragments are partitioned into independent micro-reactions, where the same index sequence is added into each sequencing fragment derived from a specific long fragment. De novo assembly of the sequencing data obtained by this method results in highly accurate genomic sequences with longer read lengths, improving accuracy and performance in detecting SVs [37, 38]. In our study, we used 10×Genomics technology to sequence the genomes of one normal female pig and two XX DSD pigs, obtaining a total of 517 Gb of clean data with an average sequencing depth greater than 60×. After de novo assembly, the genome size of all three samples exceeded 2.46 Gb, with a complete single-copy BUSCO score of more than 90%. Additionally, Jiang et al. [39] reported that the resequencing of Yorkshire pigs achieved over 99% genome coverage when the sequencing depth was 10×. These results indicate that the assembled genomes have high coverage and assembly quality, sufficient for subsequent genomic variant analysis.

A recessive inheritance pattern was applied to examine SNPs for nonsynonymous mutations, followed by screening candidate SNPs for validation in a cohort of XX DSD pigs. Our findings demonstrated that two SNPs, namely *IFITM1* (c.218T > C) and *NOBOX* (c.1043 C > G), were associated with XX DSD, yet both SNPs exhibited heterozygous mutations in the XX DSD pig cohort. Thus, the inheritance pattern of XX DSD pigs warrants further investigation through family populations. Additionally, these two SNPs only appear in XX DSD pigs and are promising molecular markers for the phenotype of XX DSD pigs.

The Interferon-induced transmembrane protein family (IFITMs) have been found to play a significant role in inhibiting cell proliferation, promoting homotypic cell adhesion, and mediating germ cell development [40]. Mouse *IFITM1*, which is expressed in primordial germ cells (PGCs), is implicated to have roles in the migration and development of PGCs [41]. The *IFITM1* gene represents a downstream target of the WNT/β-catenin signaling pathway, and interference with *IFITM1* can cause embryonic deformities [42]. WNT/β-catenin signals are necessary for normal ovarian development from the embryonic XX gonad [3]. HE staining in this study showed a lack of germ cells in the hypoplastic testes of XX DSD pigs. Previous studies have also shown that the hypoplastic testicular cords of XX DSD pigs contain germ cells at fetal stages; however, these germ cells all die within a few weeks after birth [7]. The aforementioned postnatal germ cell death phenomenon has also been seen in female mice with the *NOBOX* gene knockout [43]. Furthermore, several studies have identified *NOBOX* gene mutations as a significant cause of primary ovarian insufficiency [44, 45]. Qin et al. [46] discovered that the homozygous p.R355H mutation in the *NOBOX* gene disrupts NBE binding and affects NOBOX expression. *NOBOX* gene mutations inhibit the expression of downstream genes such as Octamer binding protein 4 (*OCT4*) and Growth differentiation factor 9 (*GDF9*), which are linked to germ cells’ development [47, 48]. Li et al. [49] discovered a homozygous truncating mutation in the *NOBOX* gene using exon sequencing, which caused G2/M phase arrest in 293FT cells. The results of this study showed that *IFITM1* mRNA was significantly highly expressed in the gonads of XX DSD pigs, whereas *NOBOX* mRNA was hardly expressed. Therefore, we hypothesise that SNPs in *IFITM1* (c.218T > C) and *NOBOX* (c.1043 C > G) may be involved in the gradual death of germ cells in XX DSD pigs by influencing gene expression. It is noteworthy that sex determination occurs during embryonic development, involving the bipotential gonad dependent on gene expression and suppression to differentiate into either testes or ovaries. Therefore, further research is needed to investigate the expression patterns of potential pathogenic genes during embryonic sex determination, in order to elucidate the relationship between gene variants and the phenotype of XX DSD pigs.

A CNV region was identified in the *SHC1* gene of XX DSD pigs by WGS analysis. As one of the transcription factors of the PI3K/AKT signaling pathway, *SHC1* is involved in oocyte maturation [50] and insulin/IGF-1-induced Ras-dependent oocyte maturation [51]. SVs validation confirmed an increased copy number of *SHC1* in only two XX DSD pigs. Further investigation is needed to understand the relationship between this CNV region and XX DSD development. The *WNT6* can activate the WNT/β-catenin signaling pathway and is involved in the growth and development of various organs and tissues, including embryonic cartilage [52], uterine [53], and spermatogenesis [54, 55]. Whole-genome sequencing detected a deletion region in *WNT6*. SVs validation showed that only two XX DSD individuals had the deletion of *WNT6*, but they also carried other SVs, including either a deletion of intron 5 of the *WWOX* gene or a deletion on chromosome 3 (spanning 231801–479100 bp). Hence, it is uncertain whether the deletion region of *WNT6* is involved in porcine DSD development. In XX DSD pigs, a deletion involving *PDGFA*, *PRKAR1B*, and *DNAAF5* on chromosome 3 spans 247,300 bp. Validation of SVs showed that 21.88% of individuals had SV polymorphisms in this region. However, the effect of this deletion remains unclear.

*SOX9* is one of the most significant genes in the XX DSD in mammals. Activated downstream of *SRY*, *SOX9* is a key player in the testiculogenesis pathway. In humans, the sex determining region (RevSex) located upstream of the *SOX9* gene has two sex reversal enhancers, Sex reversal enhancer-A and Sex reversal enhancer-B. Studies indicate that mutations in these two enhancers can lead to DSD [20]. Several studies have investigated the role of *SOX9* in pigs, to explore the molecular basis of XX DSD [8, 27, 28]. WGS analysis of XX DSD pigs identified a CNV region (8562001 ~ 8572600 bp) located downstream of the *SOX9* gene (~ 70 kb). However, validation of the SVs revealed that only 18.75% of the XX DSD pigs have this CNV, and this CNV appears in different copy numbers in the cohort. It should be pointed out that our data did not find the CNV marker region of XX DSD pigs mentioned in previous studies, which is located downstream of the *SOX9* gene (~ 500 kb) [28]. The CNV located upstream of the *SOX9* gene has been considered to cause some XX DSD cases in humans [20] and dogs [56]. Parma et al. [57] suggested that various mutations in the *SOX9* gene might lead to XX DSD in pigs. However, given that XX DSD pigs harboring the downstream CNV (8562001 ~ 8572600 bp) of *SOX9* also harboring other SVs, this CNV region alone is still insufficient to explain the pathogenesis of XX DSD pigs and needs to be validated further.

In pituitary gonadotropin cells, PITX1 regulates the synthesis of reproductive hormones and the expression of related genes, including *LHβ*, *FSHβ*, and *GNRHR* [58]. WGS sequencing results revealed a 8700 bp deletion of *PITX1*. However, the validation of SVs showed the copy number of this SV had varying degrees of increase, suggesting that *PITX1* variant is related to individual heterogeneity. Furthermore, PITX1 activates the *SOX9* promoter through a unique binding motif that induces astrocyte differentiation [59]. The synergy between PITX1 and SOX9 can also regulate skeletal development [60]. However, the regulatory role of *PITX1* on *SOX9* during sex development requires further investigation.

The *WWOX* gene encodes a WW-domain-containing oxidoreductase that acts as a transcriptional regulator and plays an essential role in various biological processes, including tumor suppression, cell proliferation, apoptosis induction, steroid metabolism, and central nervous system development [61]. WWOX expression is particularly high in the pituitary and gonads, where it is involved in the synthesis of gonadotropins and steroidal sex hormones [62, 63]. Studies on *WWOX* knockout mice have shown that *WWOX* abnormalities lead to gonadal dysfunction [64, 65], indicating its fundamental role in gonadal development. Mahmud et al. [66]also suggested a potential involvement of *WWOX* in testicular development and spermatogenesis through interaction with Golgi proteins in the Golgi apparatus of testicular cells. The association of *WWOX* with XY/XX DSD has been widely researched in humans and dogs. In humans, a heterozygous deletion spanning *WWOX* exons 6 to 8 was found to be associated with 46, XY DSD [67]. Additionally, another study documented the presence of SNP or CNV in *WWOX* exons among 46 XY DSD individuals, with SNP being identified in 46 XX DSD individual [68]. In dogs, copy number variants in exon 4 of *WWOX* were identified in XY DSD [2]. In our study, we found a heterozygous or homozygous deletion of 70 bp in intron 5 of *WWOX* gene in 62.5% of XX DSD pigs. Moreover, *WWOX* was highly expressed in the ovaries of normal female pigs and almost absent in the gonads of XX DSD pigs. This suggests that the deletion may be involved in abnormal gonadal development of XX DSD pigs. It has been previously speculated that XX DSD pigs may result from polygenic variants [6, 7]. Our SVs validation results found that of the 32 XX DSD pigs, 22 individuals contained only one SV and 10 individuals contained two or more SVs, suggesting that XX DSD may result from multiple mutations. It is noteworthy that the deletion of intron 5 of the *WWOX* gene can be used as one of the molecular markers for XX DSD pigs. We will further investigate the relationship between the 70 bp deletion in intron 5 of *WWOX* and XX DSD pigs.

## Conclusions

In sum, we found two specific SNPs, one for the *IFITM1* gene (c.218T > C) and another for the *NOBOX* gene (c.1043 C > G), found only in XX DSD pigs. It is hypothesized that these SNPs may be linked to germ cell apoptosis in XX DSD pigs. Additionally, we screened 14 candidate SVs associated with sex development, using normal sows as controls. Further validation indicated that 62.5% of the XX DSD pigs carried a 70 bp deletion fragment in intron 5 of the *WWOX* gene, which is presumed to be associated with abnormal gonadal development in XX DSD pigs. Further investigations will be carried out to delve more deeply into these findings. This study lays the foundation for studying the molecular background of XX DSD pigs and provides important reference material for studying DSD in other mammals.

## Electronic supplementary material

Below is the link to the electronic supplementary material.

Supplementary Material 1

Supplementary Material 2

## Data Availability

The data generated in this study have been submitted to the NCBI BioProject database (https://www.ncbi.nlm.nih.gov/bioproject/) under accession number PRJNA911967.
