# Supplementary material for: Whole-genome de novo sequencing reveals genomic variants associated with differences of sex development in SRY negative pigs
Source: Biol Sex Differ. 2024 Sep 2;15:68. doi: 10.1186/s13293-024-00644-w (PMC11367908; doi:10.1186/s13293-024-00644-w)
Supplement: Supplementary file 1 — Supplementary Material 1 [file 13293_2024_644_MOESM1_ESM.docx]

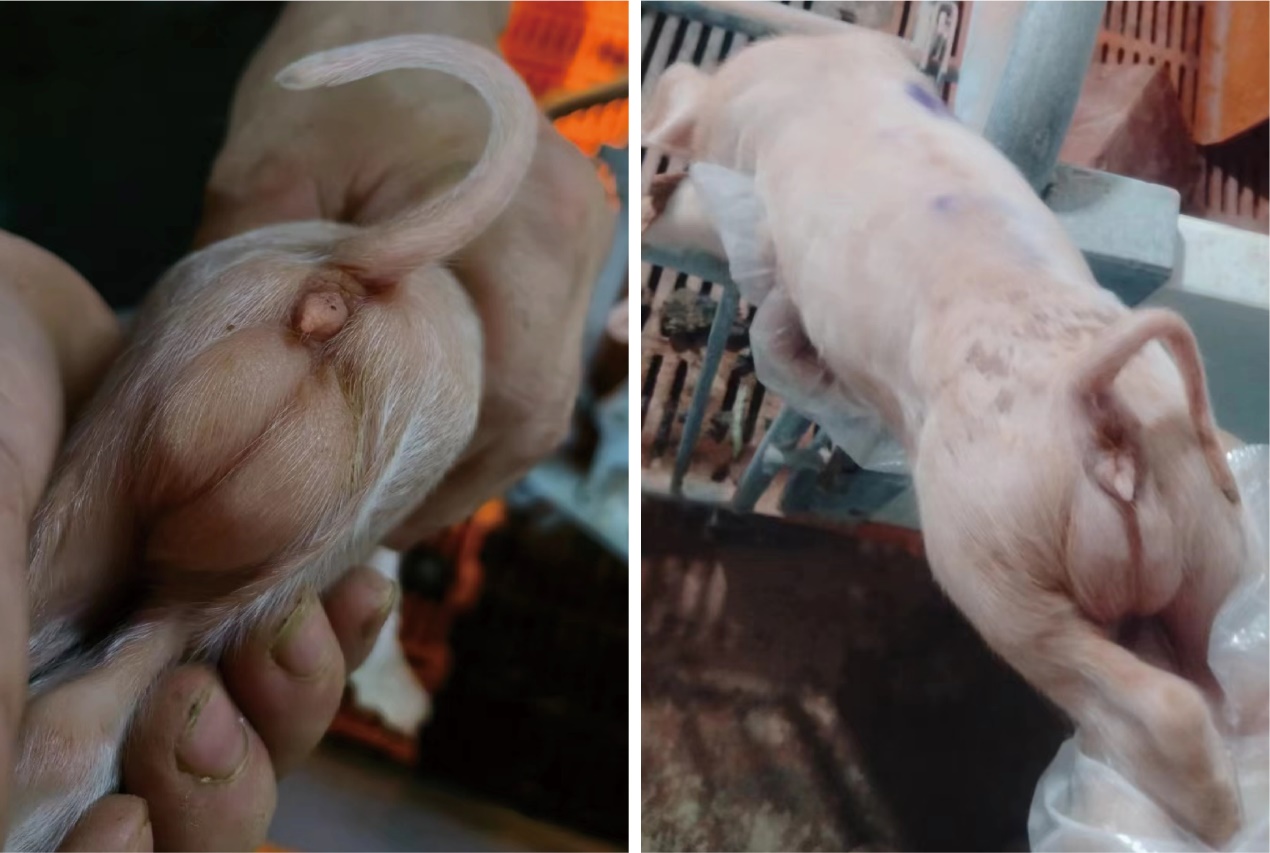


**Fig. S1** Appearance characteristics of some XX DSD pigs in the cohort study.


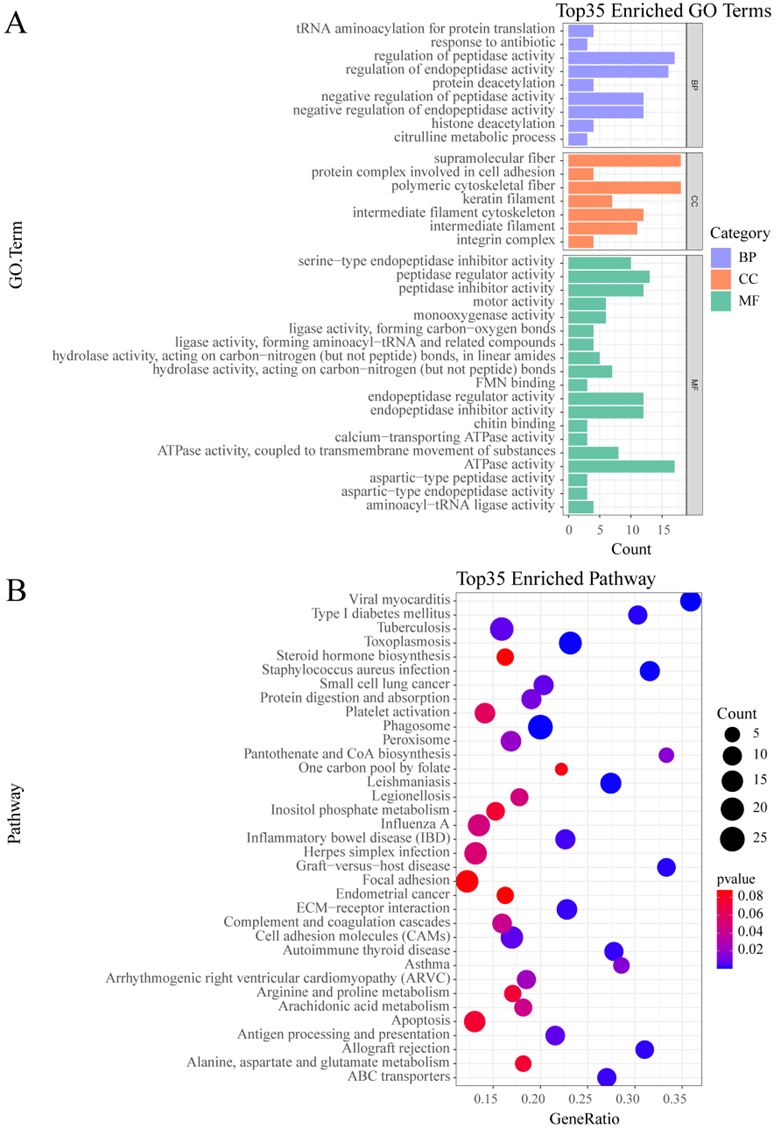


**Fig. S2** The genes harboring SNPs were analyzed using GO and KEGG enrichment. (A) Represents the top 35 enriched GO terms, BP stands for biological process, CC stands for cellular components, MF stands for molecular function. (B) Represents the top 35 enriched KEGG pathways.


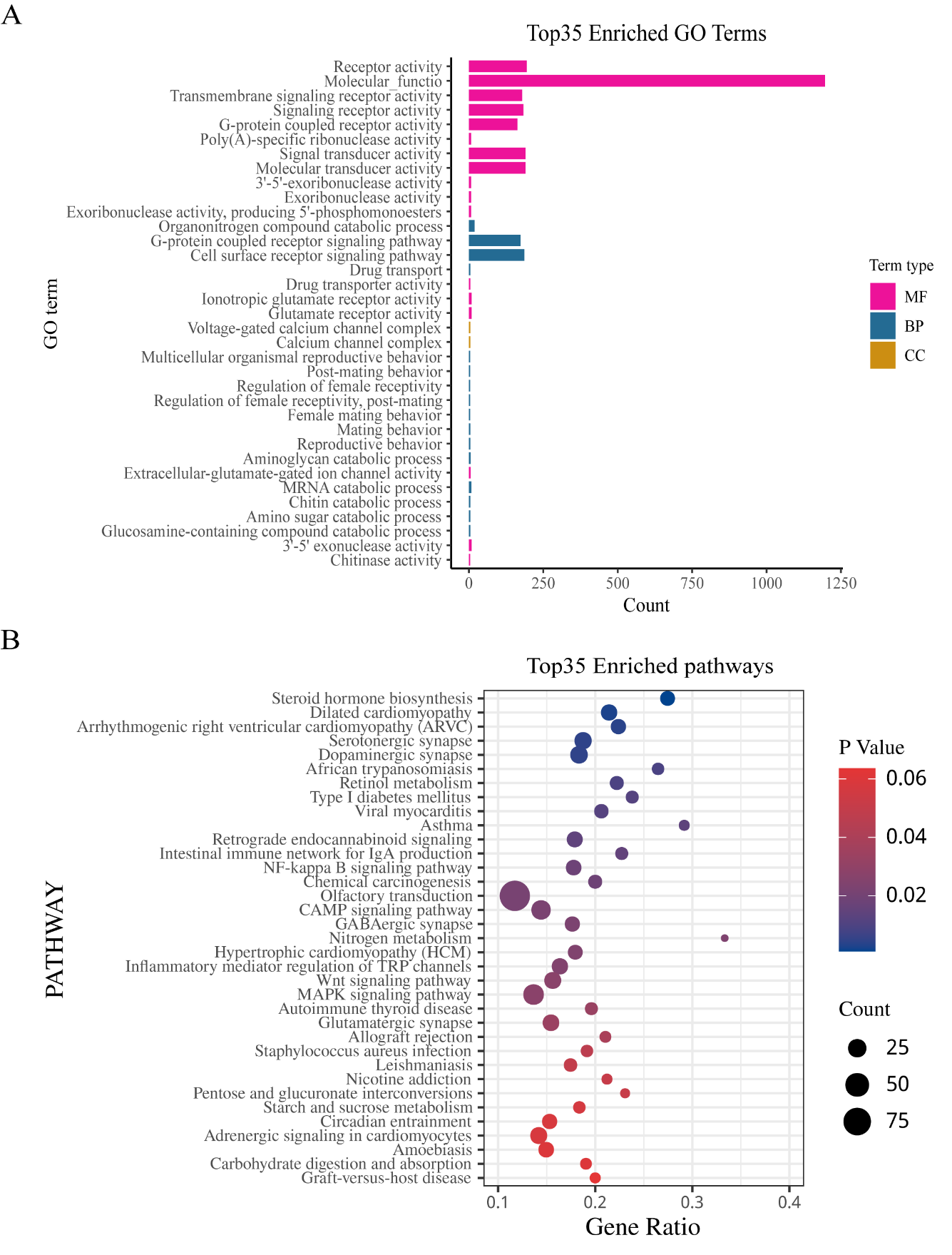


**Fig S3.** The genes harboring SVs were analyzed using GO and KEGG enrichment in XX DSD pigs. (A) Represents the top 35 enriched GO terms, BP stands for biological process, CC stands for cellular components, MF stands for molecular function. (B) Represents the top 35 enriched KEGG pathways.
