# Supplementary material for: Whole-genome de novo sequencing reveals genomic variants associated with differences of sex development in SRY negative pigs
Source: Biol Sex Differ. 2024 Sep 2;15:68. doi: 10.1186/s13293-024-00644-w (PMC11367908; doi:10.1186/s13293-024-00644-w)
Supplement: Supplementary file 2 — Supplementary Material 2 [file 13293_2024_644_MOESM2_ESM.docx]

**Table S1** Information of the duplex PCR primers for SRY

| Genes | Primer sequence | Length | Tm (℃) | Product size(bp) |
| --- | --- | --- | --- | --- |
| *SRY* | F: TGAAAGCGGACGATTACAGC | 20 | 57 | 500 |
|  | R: GGCTTTCTGTTCCTGAGCAC | 20 |  |  |
| *GAPDH* | F: CCCAGGTCTACATGTTCCAG | 20 | 57 | 311 |
|  | R: ATGGTCGTGAAGACACCAGT | 20 |  |  |

**Table S2** Primers for amplification of SNPs in candidate genes in XX DSD pigs

| Genes | Gene ID | Primer sequences | | Tm (℃) | Products (bp) |
| --- | --- | --- | --- | --- | --- |
| *IFITM1* | 100127358 | F: | GCCCTTCTGACATCCAGACACA | 59 | 542 |
|  |  | R: | ACACACCTCATGGCACATCT | 59 |  |
|  |  | F: | CTTCACCCTTGGGACTCAGAC | 58 | 590 |
|  |  | R: | GTCTCTAGCACCTGCTGTAGG | 58 |  |
| *LHCGR* | 407247 | F: | TGGTTGAGAATGGAGACTCGGC | 59 | 414 |
|  |  | R: | GCAAAACAGCAGGGAGGC | 58 |  |
| *ZFPM2* | 100337657 | F: | CCCAGAGCCAAAAGGCCAT | 59 | 284 |
|  |  | R: | CTCTGAAGCTTGAGGGACCAT | 59 |  |
| *HSD17B6* | 100620470 | F: | GGCAGATGGTTAGCCACCTT | 59 | 423 |
|  |  | R: | ACCAGCGAAGTCCTAGGTTCA | 57 |  |
| *WNT4* | 100327037 | F: | TCCACACAGCACAGGTGGA | 58 | 333 |
|  |  | R: | TGGTCAGTGGCAGCCACAA | 60 |  |
| *BMP8β* | 100620374 | F: | GGAAGGAGTTCCACTTTGACCTGA | 58 | 312 |
|  |  | R: | GGACTTGAAGCCAGGTGATACT | 58 |  |
|  |  | F: | CAGAGGAGGAAACTGAGGCAC | 60 | 334 |
|  |  | R: | CGAAGATCCCAGGCAGTTTG | 58 |  |
| *POU5F1* | 100127461 | F: | TTGATCCTCGGACCTGGCT | 59 | 298 |
|  |  | R: | AGCTTCTCCTTGTCCAGCTTC | 58 |  |
| *AMHR2* | 100154297 | F: | CTGGCCTATGAGGCAGAACTG | 58 | 187 |
|  |  | R: | TCCTGCAGACAAGCCCTTGAAG | 60 |  |
| *NOBOX* | 100499552 | F: | ATGTGCTCGTAGTCCAGGC | 59 | 247 |
|  |  | R: | ACATCAGGGCCAGCCATT | 59 |  |
|  |  | F: | TGTGGTTCCAGAATCGCCG | 59 | 287 |
|  |  | R: | AGGCACTGGCAACTTCCTAA | 56 |  |
|  |  | F: | TGCTCCACAGACCCAGCTT | 59 | 324 |
|  |  | R: | GACAATGGAACGCAAGGAACG | 59 |  |
| *LHX9* | 397597 | F: | TTGGTTCCAAAACGCACGAG | 58 | 394 |
|  |  | R: | AAGCTACACACCGAGCTGTT | 59 |  |
| *CFTR* | 403154 | F: | CCTTGGGAAGGATTCTACCATTG | 57 | 362 |
|  |  | R: | GGCTGCAGCTCTGATGCAA | 57 |  |

**Table S3** Information of qRT-PCR primers

| Genes | Primer sequence | Length | Tm (℃) | Product size(bp) |
| --- | --- | --- | --- | --- |
| *IFITM1* | F: GTGGCTTTCGCCTACTCCGT | 20 | 62 | 141 |
|  | R: AGTGGCTCCGATGGTCAGAATG | 22 |  |  |
| *NOBOX* | F: GAACCCTGTACCGCTCTGAC | 20 | 60 | 166 |
|  | R: TCCACTTTTCGCCACTTTGC | 20 |  |  |
| *GAPDH* | F: GTCGGAGTGAACGGATTTGGC | 21 | 60 | 150 |
|  | R: CTTGCCGTGGGTGGAATCAT | 20 |  |  |

Table S4 Information on the CNVplex assay for validating candidate SVs in the XX DSD pig cohort

| Fluorescent tags | Probes | Position (*Sscrofa* 11.1，realease91) | Specific primers (5’→3’) | Products (bp) | |  |
| --- | --- | --- | --- | --- | --- | --- |
| PET | ACTB_1 | 3:4084341-4084541 | F: AATAAGCGGAGCATGGGAAGC  R: AAAGACACCAGCCACTGGCAG  F: CGGTTTCAGCGCCTTGAGAA  R: GTGGCCCTCAGGTGATCAGAGT  F: GGGCCTCTGACTCTGCTCTTCT  R: TTCTGCCCAGGTGAGAGGCA  F: GTCCATCACAGCTTCTCAGCAGA  R: CAGGAGGTAGTCCAGGCTTGGTT  F: GGATGTTGGGAGCTCCTGGAGT  R: TGGTGTCCTACCAGCTCACTGTG  F: GAAGAACTGTGCCTTGGTGTTGG  R: ATGGTGGGCCTTTTATGCCTGTA  F: GTCCCATATCTCCTTTTGGTCCA  R: TATGGTCCTCTCTCCCCTGGTTT  F: GGAATCCTGAGAAAGAGGAGTGGA  R: CGTACTCAGAGGAGTACAGCCCGT  F: TTTTGACACCAGAAGGTGGAACA  R: GGGCAGGTGGAAAGAGAGTGAGA  F: GCCTGGAGTCCAGATACTTGCTGT  R: AGTCACTGGTAAACGTGCCCTGT  F: ACCAGCCAAGCAATGAATTCCTT  R: GGCAGCTTGGCCTTCCAAATAAG  F: TTTGCAGTTTCACAACTGTGGCTA  R: CCAGTCCTCCCCTTATCCTATTATTG  F: TTTGGTGACGATTCCTTTGGGTA  R: GGCCTACGTTTTCTGTCAGAGACA  F: CAAGAAAGAATTGCTCCCCATG  R: TGTAGGTTTTCCTTCAGGTTCTTCAG  F: CTGCCTTCTCTCCATGATGGTTC  R: CTTCCCTGTCCCCAGAAAATACG  F: CTTCAGCAACTCCTGCCCTGTC  R: CTCCCCAAGGGAAGGTCCTCTAA  F: CATGGGTCCTGTGTGCCTCTT  R: GTCCACCCAAGGCCCTTTATT  F: AAACACCTAAGGAACCATCCCCA  R: CAAAGGCTTTAGTCATCACAGCCC  F: TCCATTCTCAGGCTTAATGTGCAA  R: TGACCTTTGGGTACTGTGTTACAAGC  F: TCTTTTCCCAAGGAGTTCTGTAGCC  R: CGCTTTCTAGGAAATGCTGCACT  F: AGAAGGGTGCCTGGCATGAGTT  R: GCTGCTCCTTGGCATGGTAGAC  F: GTTTGAGGGGTCAGGACTGAGGT  R: GGGGCTCTACCGTTGAGCTAACT  F: GGGTGAAAATGGTCTCTGATCCC  R: TATCAGCCTCTTCCTTGCGTAAGA  F: CACACCTCTGAAATTCCCAGGTG  R: CCTTCAGGCACAAATAAGCAACC  F: AGGGGAAAGGTTTGGCATTCAG  R: ACCTTGGAGAGGTTCCTTTCCCT  F: AAGTAGGGGTTGGACTGGAGCTG  R: ATGGGAATGAACAAGGGGTGAAT  F: GAAACTCTTCCCAGATGTGCGTG  R: TCGTCCGTGTGTGGGATATGTAC  F: GTGTCAATTGGGGCATTTCATT  R: TTCCTCCCACATTGTCCATTTTATC  F: TCTTCATGGCGTAAAAGGGCAC  R: AAATGAATCACCGTCCTGTTCCA  F: CTATTCAAAAGTCCCCAGAGGCC  R: TGGTCTGTAGGAGGAGGGAGGA  F: TCTAGTATTGGCTGAGCCCTGGG  R: GCCCTGCACCCTGATTTCAG  F: GATGCAGCAAGTGAGTTGTAATCATC  R: CCCCTACCTTGACTGACACTCATTC  F: GCAATGTGTATTTCTCTTTCAACCCC  R: TACAGCTGTGTCGGACCAGGTCT  F: AGGTGATGTCTTCAGGCCAGATG  R: TCAGTCAGCTTGCAGTGACATCTG  F: TTAAAGTCTGTATTCCCTCCTGCTCA  R: TATTGCCTCCAACGACCACTTTAC  F: GGTAGGGATGGATCTTGGCTTCA  R: AGTGTGCTCAAGGCCCTTCTCTC  F: CTCATTGTTGTGGGGCCAGAAC  R: GTGCCTGGACAGCCTGTACTTGT  F: GGGACAATCTCAGTCTCTTTTGGG  R: GGATGCTGGTTGGGGAAGAG | | 94bp | |
|  |  |  |  |  |  | |
| NED | ACTB_2 | 3:4084673-4084873 |  |  | 97bp | |
|  |  |  |  |  |  | |
| FAM2 | ACTB_3 | 3:4085750-4085950 |  |  | 109bp | |
|  |  |  |  |  |  | |
| VIC | ACTB_4 | 3:4086150-4086350 |  |  | 122 bp | |
|  |  |  |  |  |  | |
| FAM2 | COL10A1_1 | 1:81773639-81773839 |  |  | 97 bp | |
|  |  |  |  |  |  | |
| PET | COL10A1_2 | 1:81772878-81773078 |  |  | 106 bp | |
|  |  |  |  |  |  | |
| VIC | COL10A1_3 | 1:81768865-81768911 |  |  | 109 bp | |
|  |  |  |  |  |  | |
| NED | COL10A1_4 | 1:81766746-81766946 |  |  | 122 bp | |
|  |  |  |  |  |  | |
| VIC | GCG_1 | 15:68810058-68810258 |  |  | 97 bp | |
|  |  |  |  |  |  | |
| NED | GCG_2 | 15:68805422-68805622 |  |  | 109 bp | |
|  |  |  |  |  |  | |
| PET | GCG_3 | 15:68803602-68803802 |  |  | 119 bp | |
|  |  |  |  |  |  | |
| FAM2 | GCG_4 | 15:68800536-68800736 |  |  | 122 bp | |
|  |  |  |  |  |  | |
| NED | 1_1 | 1:221-421 |  |  | 116 bp | |
|  |  |  |  |  |  | |
| PET | 1_2 | 1:4181-4226 |  |  | 122bp | |
|  |  |  |  |  |  | |
| PET | 2_1 | 2:137202002-137202202 |  |  | 113bp | |
|  |  |  |  |  |  | |
| NED | 2_2 | 2:137208704-137208904 |  |  | 105bp | |
|  |  |  |  |  |  | |
| PET | 3_1 | 3:232057-232257 |  |  | 98bp | |
|  |  |  |  |  |  | |
| VIC | 3_2 | 3:476955-477155 |  |  | 119bp | |
|  |  |  |  |  |  | |
| PET | 4_1 | 3:107866789-107866989 |  |  | 125bp | |
|  |  |  |  |  |  | |
| FAM2 | 4_2 | 3:107904848-107905048 |  |  | 125bp | |
|  |  |  |  |  |  | |
| NED | 5_1 | 4:94822094-94822294 |  |  | 101bp | |
|  |  |  |  |  |  | |
| FAM2 | 5_2 | 4:94825598-94825798 |  |  | 116bp | |
|  |  |  |  |  |  | |
| NED | 6_1 | 4:97372801-97373001 |  |  | 119bp | |
|  |  |  |  |  |  | |
| PET | 6_2 | 4:97376632-97376832 |  |  | 110bp | |
|  |  |  |  |  |  | |
| FAM2 | 7_1 | 4:107914842-107915042 |  |  | 113bp | |
|  |  |  |  |  |  | |
| VIC | 7_2 | 4:107922853-107923053 |  |  | 116bp | |
|  |  |  |  |  |  | |
| VIC | 8_1 | 5:185501-185701 |  |  | 113bp | |
|  |  |  |  |  |  | |
| PET | 8_2 | 5:185645-185845 |  |  | 116bp | |
|  |  |  |  |  |  | |
| PET | 9 | 6:9669013-9669213 |  |  | 102bp | |
|  |  |  |  |  |  | |
| FAM2 | 10_1 | 7:120581908-120582108 |  |  | 105bp | |
|  |  |  |  |  |  | |
| FAM2 | 10_2 | 7:120614626-120614826 |  |  | 101bp | |
|  |  |  |  |  |  | |
| FAM2 | 11 | 9:79532247-79532447 |  |  | 128bp | |
|  |  |  |  |  |  | |
| VIC | 12_1 | 9:139446384-139446584 |  |  | 125bp | |
|  |  |  |  |  |  | |
| FAM2 | 12_2 | 9:139498726-139498926 |  |  | 119bp | |
|  |  |  |  |  |  | |
| NED | 13_1 | 12:8562449-8562649 |  |  | 125bp | |
|  |  |  |  |  |  | |
| NED | 13_2 | 12:8571367-8571567 |  |  | 113bp | |
|  |  |  |  |  |  | |
| VIC | 14_1 | 15:120911408-120911608 |  |  | 105bp | |
|  |  |  |  |  |  | |
| VIC | 14_2 | 15:120913896-120914096 |  |  | 101bp | |
|  |  |  |  |  |  | |

**Table S5** Genotype frequency distribution of candidate SNPs in normal female pigs and XX DSD pigs

| Genes | SNP Site | XX DSD pigs (n=32) | | | Normal female pigs (n=32) | | |
| --- | --- | --- | --- | --- | --- | --- | --- |
| *IFITM1* | c.8G>A |  | GA (0.312) | AA (0.688) | GG (0.281) | GA (0.313) | AA (0.406) |
|  | c.139A>C | AA (0.219) | AC (0.469) | CC (0.312) |  | AC (0.531) | CC (0.469) |
|  | c.227T>C |  | TC (0.219) | CC (0.781) |  | TC (0.719) | CC (0.281) |
|  | c.320C>T |  | CT (0.656) | TT (0.344) |  | CT (0.844) | TT (0.156) |
|  | c.359G>A | GG (0.594) | GA (0.218) | AA (0.188) | GG (0.344) | GA (0.375) | AA (0.281) |
|  | c.348T>G | TT (0.750) | TG (0.125) | GG (0.125) | TT (0.531) | TG (0.375) | GG (0.094) |
|  | c.368G>A |  | GA (0.781) | AA (0.219) |  | GA (0.625) | AA (0.375) |
| *LHR* | c.26G>A | GG (0.594) | GA (0.344) | AA (0.062) | GG (0.719) | GA (0.250) | AA (0.031) |
|  | c.70A>G |  | AG (0.562) | GG (0.438) |  | AG (0.406) | GG (0.594) |
| *ZFPM2* | c.1333T>C |  | TC (0.719) | CC (0.281) | TT (0.219) | TC (0.562) | CC (0.219) |
| *HSD17B6* | c.275C>T |  | CT (0.688) | TT (0.312) |  | CT (0.562) | TT (0.438) |
|  | c.151G>C | GG (0.313) | GC (0.406) | CC (0.281) | GG (0.500) | GC (0.375) | CC (0.125) |
| *WNT4* | c.861C>A | CC (0.813) | CA (0.187) |  | CC (0.438) | CA (0.375) | AA (0.187) |
| *BMP8B* | c.487G>A | GG (0.812) | GA (0.188) |  | GG (0.625) | GA (0.219) | AA (0.156) |
|  | c.767C>T | CC (0.438) | CT (0.437) | TT (0.125) | CC (0.656) | CT (0.281) | TT (0.063) |
|  | c.691G>A | GG (0.406) | GA (0.594) |  | GG (0.563) | GA (0.437) |  |
|  | c.682G>A | GG (0.813) | GA (0.187) |  | GG (0.781) | GA (0.219) |  |
| *POU5F1* | c.169G>A | GG (0.875) | GA (0.125) |  | GG (0.781) | GA (0.219) |  |
| *AMHR2* | c.1472G>A | GG (0.406) | GA (0.563) | AA (0.031) | GG (0.281) | GA (0.563) | AA (0.156) |
| *NOBOX* | c.131G>A | GG (0.594) | GA (0.250) | AA (0.156) | GG (0.469) | GA (0.375) | AA (0.156) |
|  | c.781G>T |  | GT (0.750) | TT (0.250) | GG (0.281) | GT (0.344) | TT (0.375) |
|  | c.1103C>A | CC (0.625) | CA (0.219) | AA (0.156) | CC (0.469) | CA (0.437) | AA (0.094) |
| *LHX9* | c.1114G>A | GG (0.844) | GA (0.094) | AA (0.062) | GG (0.719) | GA (0.219) | AA (0.062) |
|  | c.1115T>C |  | TC (0.656) | CC (0.344) | TT (0.406) | TC (0.469) | CC (0.125) |
| *CFTR* | c.80C>T | CC (0.594) | CT (0.187) | TT (0.219) | CC (0.438) | CT (0.375) | TT (0.187) |

**Table S6** Copy numbers information for six SVs from 32 normal female pigs

| SVs | Type | Genomic Position | Copy numbers | | | |
| --- | --- | --- | --- | --- | --- | --- |
|  |  |  | 1 | 2 | 3 | 5 |
| *PITX1* | Deletion | 2:137200501-137209200 | 0 | 31 | 0 | 1 |
| *PDGFA/PRKAR1B/DNAAF5* | Deletion | 3:231801-479100 | 1 | 31 | 0 | 0 |
| *SHC1* | Duplication | 4:94822101-94826100 | 0 | 32 | 0 | 0 |
| *SOX9* | Duplication | 12:8562001-8572600 | 0 | 31 | 1 | 0 |
| *WNT6* | Deletion | 15:120911501-120914400 | 0 | 32 | 0 | 0 |
| *WWOX* | Deletion | 6:9669113-9669182 | 2 | 30 | 0 | 0 |

Note：*SOX9* stands for CNV in the region downstream of the *SOX9* gene.
